# Supplementary material for: Under the knife: Unfavorable perceptions of women who seek plastic surgery
Source: PLoS One. 2021 Sep 7;16(9):e0257145. doi: 10.1371/journal.pone.0257145 (PMC8423238; doi:10.1371/journal.pone.0257145)
Supplement: Attachment — Submitted filename: Plastic Surgery - Plos One.docx [file pone.0257145.s001.docx]

Overall

This is an interesting and novel research. The widespread of plastic surgery in modern society means that such a study is needed and can aid our understanding of psychological issues involved with both those eyeing plastic surgery and their networks as well as the general public.

The research design is well constructed. The variables are generally clear and derived from well explained psychological theories. The authors also show a good understanding of the literature surrounding the topic, though such literature is currently limited. This shows clearly how the research fills some knowledge gaps.

The findings are well presented overall and the discussion good.

However, there are some areas that require clarification and some adjustments.

1. Abstract and Introduction: Here I suggest that the authors provide some definition of the terms ‘attractiveness’ and ‘unattractiveness’. If these are major construct unpinning the perceptions that they are seeking to explain, then they should describe what they mean by these terms. It may be that they see attractiveness and unattractiveness from the participants’ subjective appreciation/judgement, but then the authors have to explain that.
2. Introduction (p.3, line 36). The authors claim that plastic surgery demand and operations have increased significantly. It will be helpful if they provide some data from the literature or health to substantiate this. Also, they could define a broad time period over which such increase can be observed.
3. Hypotheses (p.9). Overall, it would be helpful if the authors number their hypotheses for ease of reference from a reader perspective but also for themselves to recapture them in the discussion or conclusion. The hypotheses on the same p.9 (line…) is not clearly related to the research question and the aim of the study which to examine the perception of women seeking plastic surgery or having had plastic surgery. I do see the connection. Perhaps the authors could explain and establish the link clearly.
4. Methods (p.10). The word [in] might be missing before [total] in the following phrase (i.e. 16 photographs total). Same for line 224 on p.11 in the phrase (of the 16 total) and line 270 on p.13 in the phrase (16 items total). Please check these parentheses.
5. Results. On p.20, the coverage of Justice sensitivity needs elaboration drawing of the data and contrasting with the literature.
6. Implications. The authors could elaborate on the practical implications of the study under the heading on p.20.
7. Future research. I think it would interesting for future research to also look at the consequences of such negative perception of ‘attractive’ women undergoing plastic surgery. Does it lead to physical aggression? Stereotyping? Some forms of exclusion? And what those women affect could do?

I hope these suggestions are helpful to the authors in revising this interesting paper.
