# Supplementary material for: Under the knife: Unfavorable perceptions of women who seek plastic surgery
Source: PLoS One. 2021 Sep 7;16(9):e0257145. doi: 10.1371/journal.pone.0257145 (PMC8423238; doi:10.1371/journal.pone.0257145)
Supplement: Attachment — Submitted filename: Response to Reviewers.docx [file pone.0257145.s002.docx]

**Reviewer 1**

**R1.1** **Miscellaneous**

**Although it was highlight, that perceptions of attractive women are worsened when these women decide to seek cosmetic surgery, authors only refer that psychoeducation content can be developed for women considering plastic surgery, without specifying which ones.**

We have made clearer throughout our manuscript the practical implications that may arise from our findings. Namely, we have shifted focus away from the generation of psychoeducation content. Instead, we focus on how the present findings might shape our understanding of plastic surgery attitudes, and how women who might be considering plastic surgery can benefit from our findings. We feel these implications are more realistic and more in line with the aims of the present study. See changes throughout our manuscript and see below for an example of these changes.

e.g., “*This study is one of few that elucidates the potential psychosocial challenges women seeking plastic surgery might face. As such, findings from this study might prove informative over and above any information women might receive from surgeons or surgical clinics (i.e. where the focus in on physical outcomes only) and/or might work to educate surgeons themselves so that they are better able to inform their clientele. Specifically, we feel that from an ethical standpoint it is important for women seeking cosmetic surgery to fully understand the implications of their decisions (e.g., in undergoing cosmetic surgery, they may be perceived as less human, among other things).” p25*

**R1.2** **Title**

**Should be written in sentence case (only the first word of the text, proper nouns, and genus names are capitalized).**

We applied this revision.

*“Under the knife: Unfavorable perceptions of women who seek plastic surgery”* *p1*

**R1.3 Abstract**

**R1.3.1 Line 15: “….985 adults…” – what kind of sample (random, convenience)? Please explain.**

We have specified that our sample was random.

*“We presented a random sample of 985 adults (male = 54%, M_age_ = 35.84 years) recruited via Amazon’s Mechanical Turk with a series of experimental stimuli” p2*

**R1.3.2 Line 16: “…M age…” Please add the standard deviation.**

We have added standard deviation

*“(male = 54%, M_age_ = 35.84 years, SD_age_ = 10.59)” p2*

**R1.3.3 Line 19: “…humanness”. In the end of the sentence, please refer the statistical analyses performed.**

We have included our analysis.

*“Participants rated stimuli on perceived warmth, competence, morality, and humanness. We ran linear mixed-effect models to assess all study hypotheses.”p2*

**R1.3.4 Line 21: “… control activities …” Please specify/give examples.**

We feel that adding more information pertaining to the control activities in the abstract would confuse readers more so than aid clarity. However, we have added an additional example of for control activities in the Materials and Measures section of our Method. An exhaustive list of our control vignettes has now also been uploaded to the OSF, and this has been indicated in our manuscript.

*“Of these, 12 were control vignettes that depicted a woman planning to undergo a neutral – that is, common or everyday – activity (e.g., “this woman is planning to eat a meal”, “this woman is planning to buy a pet”). The remaining vignette was our plastic surgery target vignette – “this woman is planning to have plastic surgery”. Again, each participant would read four of these vignettes in total; three control vignettes (of the 12 in total) and the plastic surgery vignette. Further details are outlined in the procedure. A full list of control vignettes can be found on the Open Science Framework (*[*https://tinyurl.com/ska2qv9)*](https://tinyurl.com/ska2qv9))*.” p11*

**R1.4 Introduction**

**R.1.4.1 Despite being presented, in theoretical terms, the contextualization of the problem, it is not elaborated, in practical terms, what this investigation can contribute to the resolution of the problem. Please explain to what extent the perception of others about plastic surgery is important and how it may have practical implications for those who intend to perform plastic surgery.**

See our response to R1.1 for an overview of how we included more practical implications in our discussion. For our introduction, we also included this line:

*“This knowledge will help contribute to our growing understanding of how society perceives plastic surgery, thereby elucidating the challenges women who choose to undergo it are likely to face. Without this study, uninformed women may choose to seek out plastic surgery and find themselves surprised and disappointed by the negative psychosocial consequences they face postoperatively.”* *p5*

**R1.4.2 Line 67: “…Brazilian women”. Please cite reference.**

We have added three references.

*“…power for Brazilian women; 13–15).” p4*

**R1.4.3 Line 84 – “…psychoeducation content…”: Please elaborate / specify.**

This line has been removed from the manuscript, rendering this suggestion obsolete. See R1.1 for removal justification.

**R.1.3.4 Lines 173-175; 177-178: Very confusing phrases: after all you intend to evaluate the perception of women who intend to have a plastic surgery or the perception of others about women who intend to have plastic surgery? Please clarify since it is incongruous with the presented hypotheses.**

We have reworded these lines.

*“In an attempt to examine the social consequences of undergoing plastic surgery, the present paper will therefore examine how women who seek plastic surgery are perceived across four domains: warmth, competence, morality, and humanness … We aimed to examine whether perceptions of women who seek plastic surgery systematically differ from perceptions of women who do not.”p9*

**R1.3.5 Since the participants were from the two sex, it would be interesting to explore if women seeking plastic surgery are perceived by males and females in the same way.**

We examined the effect of participant gender on the negative plastic surgery effect. While effects were non-significant for most outcome variables (warmth, competence, and humanness), we did find that plastic surgery status only predicted *morality* scores in cases where participants were female. In other words, women seeking plastic surgery were only considered less moral than those not seeking surgery by female participants.

While ultimately we did not decide to include this exploratory analysis in our revised manuscript (given that it largely produced non-significant results and was not part of our initial analysis plan), we do value improving our understanding of these variables and their interactions and thank the reviewer for their suggestion.

**R1.5 Material and Methods**

**R1.5.1 Line 196: Explain how the sample size was calculated. If a sample size calculation was performed, specify the inputs for power, effect size and alpha.**

No sample size calculation was performed for this study. In hindsight, we understand that ideally a sample size calculation would have been performed. However, in the interest of transparency, we note that sample size was informed exclusively by funding available to our lab at the time of data collection. We have indicated this in the manuscript.

*“Sample size was determined prior to data analysis based on funding available to our research team at the time of data collection.”p10*

**R1.5.2 Lines 202-203: ”… see …data quality). This sentence should be cut and only the reference should appear. In limitation you should refer this.**

We have amended this sentence. We do not believe our compensation to be a limitation; rather, we were attempting to refer readers to this article in case they were wondering why we compensated participants more generously than most other studies on MTurk.

*“Compensation for each participant completing our 15-minute survey was US$2.33 (42).”p10*

**R1.5.3 Lines 206-207: ”… see …checks). This sentence should be cut and only the reference should appear. Justify in the text, why you excluded 16 participants and cite the reference.**

We have amended this.

*“We excluded 16 participants from the present study for failing to consistently report their race. To elaborate, we included two items in our survey asking participants to indicate their race – one toward the commencement, and one toward the conclusion, of the survey. In cases where participant responses did not align between these two items, ‘participants’ were assumed to be bots (or to simply not be paying attention) and were subsequently removed from the study (43).” p10*

**R1.5.4 Line 227: How many participants for the pilot study?**

208. We have included this in the manuscript. This information is also available in the data file on the Open Science Framework.

*“All vignettes were assessed for their ability to induce participant affect and arousal in an MTurk pilot study (N = 208)” p11*

**R1.5.5 What is the assumptions criterion used for LMM?**

Before interpreting the output of any multilevel models, we checked the key assumptions of linearity of relationships (where relevant, as many of our analyses had binary predictors only, thus linearity was inherent), normality of residuals, and homoscedasticity. For these checks, we took a graphical approach as recommended by Fife (2020). We used the R-packagesjPlot (Lüdecke,2020) to draw diagnostic plots. To check for linearity, we plotted model residuals against predictors. To check for normality in fixed effects, we plotted a Q-Q plot of residuals and the distribution of residuals. To check for normality in random effects, we plotted random effect quantiles against standard normal quantiles. To check for homoscedasticity, we plotted fitted values against residuals. All assumptions fell within reasonable bounds upon visual inspection.

**R1.5.6 Describe any analysis carried out to confirm the data meets the assumptions of the analysis performed (e.g.: linearity, co-linearity, normality of the distribution).**

See R1.5.5

**R1.5.7 Please explain in detail how was made the data generation (Describe the technical details or procedures required to reproduce the analysis)**

This is available on the Open Science Framework. We had noted this in our manuscript, but have also amended the wording to make it clear that a full guide is provided on the Open Science Framework.

*“All data and a step-by-step guide for our statistical analyses (including data cleaning) are available on the Open Science Framework (*[*https://tinyurl.com/ska2qv9*](https://tinyurl.com/ska2qv9)*).”p15*

**R1.5.8 Please indicate what was the statistical package software used to carry out LMM analyses (List the name and version of software package used, alongside any relevant references).**

RStudio Version 1.2.1335 was used.

*“All analyses were conducted in RStudio Version 1.2.1335 (48).”p15*

**R1.6 Results**

**R1.6.1 Lines 313-314: “...these interactions). Please indicate the ß and CI 95%.**

ß and CI 95% can be calculated using the step-by-step guide we provide on the Open Science Framework. We feel as though providing all ßs and CIs in our manuscript would add unnecessary confusion and bulk to our text. However, we have added a note in our manuscript that these statistics are accessible using the Open Science Framework.

*“Participant scores for justice sensitivity and disgust sensitivity did not moderate any of these interactions. Betas and confidence intervals for these non-significant interactions are accessible using our step-by-step guide on the Open Science Framework (*[*https://tinyurl.com/ska2qv9)*](https://tinyurl.com/ska2qv9))*.” p15*

**R1.6.2 Line 326: “…were also were…” cut the second “were”.**

We’ve cut out this typo.

**R1.6.3 Lines 340-341: “...these relationships). Please indicate the ß and CI 95%.**

See R1.6.1

*“However, participant scores for justice sensitivity and disgust sensitivity did not moderate these relationships. Betas and confidence intervals for these non-significant interactions are accessible using our step-by-step guide on the Open Science Framework (*[*https://tinyurl.com/ska2qv9)*](https://tinyurl.com/ska2qv9))*.” p18*

**R1.7 Discussion**

**R1.7.1 Lines 349-350: Again, very confusing phrase: you intend to evaluate the perception of women who intend to have a plastic surgery or the perception of others about women who intend to have plastic surgery? Please clarify**

Again, we have amended our wording.

*“The present study built on existing literature by examining whether women seeking plastic surgery are systematically perceived differently to other women.” p19*

**R1.7.2 Line 353: “…control activities”: Please give examples.**

Given that we have reiterated that control activities can be found on the Open Science Framework, as well as included more examples in our method section (see R1.3.4), we don’t feel it necessary to here again include examples of control activities. However, if the reviewer ultimately disagrees, we will happily amend to include the discussion to include examples.

**R1.7.3 Line 369: “…is unique” Please cite references.**

We have included a reference for this sentence.

*“…has demonstrated that the way in which people relate to members of each quadrant is unique (37).” p19*

**R1.7.4 Lines 376-377: “…seeking plastic surgery is likely to foster implication for women …”. I wonder, although this is a possibility in the face of the choice of a woman to decide to have plastic surgery, something that in principle should only concern her, the fact that she does not do it for the sake of others, will have milder or more serious psychosocial implications/impact for the own? Please elaborate about it.**

We agree that it would be interesting to investigate whether women’s motivations for undergoing surgery (i.e. for themselves versus for others) would affect how the negative plastic surgery effect might impact their psychosocial wellbeing. However, because this area of research is so underexamined, we feel there are an abundance of potential, unexplored variables that might moderate this relationship (e.g., surgical outcome, recipient gender, recipient age etc). As such, we feel it is outside the scope of our paper to address all of these moderators, and would prefer not to incorporate one in lieu of others.

**R1.7.5 Line 385: “…inform our own”. Please cite reference.**

This line is already cited – perhaps this was an accidental oversight by the reviewer.

*“In line with socialization theory (whereby the beliefs of those around us heavily inform our own), we induce that women considering plastic surgery may struggle to ‘feel moral’ if others perceive them to be morally questionable (56).” p20*

**R1.7.6 Line 387: “…wellbeing relative to …”. Relative to what?? Please explain.**

Again, we believe this might be a reviewer oversight, as we explain this is relative to if they were not seeking plastic surgery.

*“In turn, they may experience worsened wellbeing relative to if they were not seeking plastic surgery.” p20*

**R1.7.7 Line 394: “…and that this in turn …”. Cut “that this”.**

We believe the original wording of the sentence to be grammatically correct, but added a comma for clarity.

***“****Overall, we conclude that the ability to fulfil psychological needs may be affected for women seeking plastic surgery, and that this in turn may detriment their wellbeing.” p20*

**R1.7.8 Lines 400-401: “…was not …by results”. Why? What is the possible explanation? Please elaborate.**

We have expanded on this section. See R2.5.

**R1.7.9 Lines 417-419: “As such, …surgery effect”. Why? What is the possible explanation? Please elaborate.**

Ultimately, given that this was a purely exploratory hypothesis grounded in little existing literature or theory, we have decided not to expand on our interpretation of this result. However, we are happy to make further changes and expand on this section if the reviewer is not happy with our decision.

**R1.7.10 Lines 426, 435, 449: “To elaborate”. Please avoid the systematic repetition of the expression.**

We removed one of these sentences from our manuscript, and reworded another to avoid repetition.

**R1.7.11 Lines 460-463: The author refers to women perception who intend to have a plastic surgery or the perception of others about women who intend to have plastic surgery? Please clarify!**

We have reworded this sentence to aid clarity.

*“In other words, while there were statistically significant differences between perceptions of women who seek surgery and women who do not across all outcome measures, the absolute difference in perceptions of these women were minimal.” p24*

**R1.7.12 Limitation: As you refer on the participants section, it was made a compensation for each participant completing the survey. Taking this in account, how do you guarantee honest responses?**

Providing compensation to participants is generally considered best practice, so long as payment amount is appropriate (i.e. not so large that it is deemed coercive or undue; e.g., see Ripley [2006] for a review). While we cannot guarantee honest responses, we did include an attention check measure in our study to ensure ‘bots’ and distracted workers’ responses were not included in our analyses (see R1.5.3).

**R1.7.13 Line 471: “…psychoeducation content…”. Please elaborate / specify.**

We have removed this line. See R1.1.

**R1.7.14 Lines 473-475: “… we suspect…planning to do so”. I am confused… taking in account that this study “… posits that exploring plastic surgery as an intended action would enable researchers to better assess negative perceptions …rather than its associated outcomes”, how do you explain your suspicious??**

We agree with the reviewer’s concerns regarding this section, and have subsequently removed it from our discussion.

**R1.7.15 Lines 478-479: “Overall…experimentally”. By whom?? Please refer.**

We suspect the reviewer has misread this sentence in our manuscript, as we are referring to our own study at this point (see below). However, we are happy to revisit the comment if we have misunderstood.

*“Overall, our study was the first to examine the negative plastic surgery effect experimentally.” p25*

**R1.7.16 Lines 479-480: “we…attitudes”. Please highlight the importance of the thematic, concerning future studies.**

We weren’t too sure what the reviewer meant by ‘thematic’, but we have attempted to address this feedback by including more information about future implications.

*“We provide a fundamental starting point from which future literature can further investigate negative plastic surgery attitudes in order to inform both women seeking cosmetic surgery and cosmetic surgeons themselves.” p25*

**R1.8 References/Citations**

**If the intention is to use Vancouver style consistently, please revise its correct use and format throughout the text. Special attention should be given to citations of references (e.g., Lines: 56, 74, 100, 124, 135, 151, 163, 203 (this one is in APA style), 207, 215…).**

**Please revise “DOI” number and adhere to the format give in “PLOS ONE Submission Guidelines”.**

We have removed page numbers (e.g., line 56). For lines 74, 100, 124, 135, 151, 163, and 215, bracketing has been corrected. We have amended lines 203 and 207. Further, as per PLoS ONE guidelines, DOI numbers can be used in place of page numbers but are not necessary for inclusion if page numbers are already included. As such, we have not added DOIs to our references.

**R1.9 Tables**

**The titles should not be in italic and should be align with table identification.**

We have amended this formatting.

**Reviewer 2**

**R2.1 Abstract and Introduction: Here I suggest that the authors provide some definition of the terms ‘attractiveness’ and ‘unattractiveness’. If these are major construct unpinning the perceptions that they are seeking to explain, then they should describe what they mean by these terms. It may be that they see attractiveness and unattractiveness from the participants’ subjective appreciation/judgement, but then the authors have to explain that.**

We have added a brief note in our introduction to make clear what we mean by attractiveness. Also, we have reiterated how attractiveness was conceptualised in the present study in the materials and measures section of our method.

*“An individual’s physical attractiveness – by which we mean their average attractiveness score as rated by others – affects how others perceive them.” p5*

*“We compiled a smaller database containing the eight most and eight least attractive (as rated by the 1087 CFD participants) White women contained in the CFD for use in the present study (i.e. 16 photographs in total).” p11*

**R2.2 Introduction (p.3, line 36). The authors claim that plastic surgery demand and operations have increased significantly. It will be helpful if they provide some data from the literature or health to substantiate this. Also, they could define a broad time period over which such increase can be observed.**

We agree, and have made the necessary revisions.

*“Today, approximately 18 million plastic surgeries are performed in the US each year – a 169% increase since the year 2000 (2,3). Further, rates increased by more than 20% worldwide between the years 2015 and 2019 (4).” p3*

**R2.3** **Hypotheses (p.9). Overall, it would be helpful if the authors number their hypotheses for ease of reference from a reader perspective but also for themselves to recapture them in the discussion or conclusion. The hypotheses on the same p.9 (line…) is not clearly related to the research question and the aim of the study which to examine the perception of women seeking plastic surgery or having had plastic surgery. I do see the connection. Perhaps the authors could explain and establish the link clearly.**

We have numbered our hypotheses (1, 2, 3, 4a, 4b) for ease of reference. We were not entirely sure whether the editor’s comments pertaining to ‘the hypotheses on the same p.9 (line…)’ referred to a specific hypothesis on p9, or rather our hypotheses in general. However, we posit that all our hypotheses *bar hypothesis 3* are directly related to examining the perception of women seeking plastic surgery. Therefore, we assume the reviewer is referring to hypothesis 3. In light of this, we contend that hypothesis 3, while not directly related to our manuscript’s research question, works toward validating existing literature (as stated in the manuscript). Therefore, we feel its inclusion is justified. We hope this response has satisfied the reviewer’s concerns and are happy to make future changes to the manuscript if necessary.

*“Our primary hypothesis was that (1) women seeking plastic surgery would be considered less warm, competent, moral, and human than those who are not (i.e. there would be a negative plastic surgery effect). We also hypothesized that (2) this relationship… (3)… (4a)… (4b)”p9*

**R2.4 Methods (p.10). The word [in] might be missing before [total] in the following phrase (i.e. 16 photographs total). Same for line 224 on p.11 in the phrase (of the 16 total) and line 270 on p.13 in the phrase (16 items total). Please check these parentheses.**

We have applied these revisions to all noted parentheses.

**R2.5 Results. On p.20, the coverage of Justice sensitivity needs elaboration drawing of the data and contrasting with the literature.**

We have elaborated in this section.

*“Firstly, contrary to existing literature, these findings do not support the theory that the negative plastic surgery effect is driven by concerns pertaining to recipients reaping ‘unearned’ rewards from plastic surgery (i.e. the concern that plastic surgery is 'cheating'; 11,25,55). Further, also in contrast with existing literature, these findings also do not support the theory that the negative plastic surgery effect is driven by stigmatization toward individuals with non-normative bodies (22,26,27). We note, however, that because of our study design, stimuli described as having had plastic surgery in the present study did not necessarily look non-normative (i.e. they did not look different to control stimuli). As such, we cannot definitively conclude that perceptions of disgust do not drive the negative plastic surgery effect in cases where women have visibly undergone surgery (e.g., they look ‘artificial’); rather, only in cases where plastic surgery status alternatively becomes known.” p21*

**R2.6 Implications. The authors could elaborate on the practical implications of the study under the heading on p.20.**

See R1.1. We have incorporated our implications into a section titled “Implications, Conclusions, and Future Directions” on page 24.

**R2.7 Future research. I think it would interesting for future research to also look at the consequences of such negative perception of ‘attractive’ women undergoing plastic surgery. Does it lead to physical aggression? Stereotyping? Some forms of exclusion? And what those women affect could do?**

We have incorporated this suggestion in the final section of our manuscript.

*“Future research might also explore additional consequences that women subject to the negative plastic surgery effect are likely to face. For example, might this worsened person perception result in the social exclusion or prejudicial treatment?”* p24

**Reviewer 3**

**The premise of this research and the methods used (i.e. rating women on attractiveness) is steeped in misogyny and has no place in contemporary scholarship. We must do better. See this media piece for a summary of the types of issues that are promoted by your work: https://www.theguardian.com/society/2020/aug/05/disgusting-study-rating-attractiveness-of-women-with-endometriosis-retracted-by-medical-journal**

We appreciate and share reviewer 3’s disdain for the ongoing misogyny rife in contemporary scholarship. Further, we are familiar with the endometriosis study provided as an example by reviewer 3 and also believe this work to be fundamentally problematic. However, we disagree with reviewer 3 with regards to the role our own manuscript plays in promoting misogyny in contemporary research. Notably, while we (assumedly) agree with reviewer 3 in that women *should* not be judged based on their attractiveness, we feel it is important to acknowledge that they nevertheless *are* (see Dion’s 1972 seminal “What is beautiful is good”; see commentary on ‘lookism’ by Australian scholar Beatrice Alba – <https://tinyurl.com/4fd4azbp>). The intent of our manuscript is not to encourage the objectification of women, but rather (in part) to investigate the very real and important role attractiveness plays in person perception.

Dissimilarly to the endometriosis study referenced by reviewer 3, we feel our manuscript has clear scientific merit. For example, our study works to validate existing literature as well as anecdotal accounts of women’s lived experiences of discrimination based on ‘lookism’ (see commentary above). Further, our study importantly examines whether women seeking cosmetic surgery – potentially to avoid facing discrimination based on their attractiveness – might actually be subject to *further* discrimination for choosing to undergo surgery. Finally, we note that at no stage during our study were participants actually asked to rate each other (or even photographed stimuli) on attractiveness. Rather, our methodology relied on a set of pre-existing, validated faces already rated on attractiveness (the Chicago Faces Database; CFD). Importantly, CFD participants provided full informed consent to be rated on attractiveness.

Lastly, we would like to make known to reviewer 3 that all collaborators on this paper are members of what we consider to be a feminist research team. While we may not agree with reviewer 3’s concerns regarding our work, we very much do appreciate the opportunity to engage in feminist dialogue and recognise that differences in opinion will exist even within the feminist community. In an attempt to placate some of reviewer 3’s concerns, we have added a brief note in our introduction to make clear what we mean by ‘attractiveness’ in this context (thereby eliminating any misconceptions that our research team believes beauty to be an entirely objective or dichotomous construct). Further, we have reiterated how attractiveness was conceptualised in the present study in the materials and measures section of our method. We hope that this response has addressed reviewer 3’s concerns; however, we are of course happy to take on board any further suggestions for improving our manuscript.

*“An individual’s physical attractiveness – by which we mean their average attractiveness score as rated by others – affects how others perceive them.” p5*

*“We compiled a smaller database containing the eight most and eight least attractive (as rated by the 1087 CFD participants) White women contained in the CFD for use in the present study (i.e. 16 photographs in total).” p11*
