# Supplementary material for: Under the knife: Unfavorable perceptions of women who seek plastic surgery
Source: PLoS One. 2021 Sep 7;16(9):e0257145. doi: 10.1371/journal.pone.0257145 (PMC8423238; doi:10.1371/journal.pone.0257145)
Supplement: Attachment — Submitted filename: Plastic Surgery - Pos One -2.docx [file pone.0257145.s003.docx]

The authors have answered most of my comments. However, some minor issues remain. These are listed below:

Line 13, on p. 2: The authors should provide some literature evidence to support the assertion that [plastic surgery is increasing in popularity]

Line 157, on p.8: “First proposed in 2002, the Stereotype Content Model proposes”. Stereotype Content Model should be referenced.

Line 159, on p.8: The phrase “the way we feel about others is said to depend” should be referenced. Who says this?

Line 174 on p. 9: The word “therefore” is redundant.

Line 315 on p.15: The word “Results” should be preceded by [The]

Line 326 on p. 16: “They were also were rated…” one [were] is redundant
