# Supplementary material for: Under the knife: Unfavorable perceptions of women who seek plastic surgery
Source: PLoS One. 2021 Sep 7;16(9):e0257145. doi: 10.1371/journal.pone.0257145 (PMC8423238; doi:10.1371/journal.pone.0257145)
Supplement: Attachment — Submitted filename: Response to Reviewers.docx [file pone.0257145.s004.docx]

**Response to Reviewers**

**R1.1** **Line 258 -259: Please put in () the sentence “ A full list…”.**

*“Again, each participant would read four of these vignettes in total; three control vignettes (of the 12 in total) and the plastic surgery vignette (vignettes can be accessed on the Open Science Framework;* [*https://tinyurl.com/ska2qv9*](https://tinyurl.com/ska2qv9)*). Further details are outlined in the procedure.” p11*

**R1.2** **Statistical Analyses: Include in the text, the assumptions criterion used for LMM, according to your reviewer answer.**

We appreciate the reviewer’s suggestion to provide assumption checking criteria. Ultimately, we felt it more appropriate to include these criteria on the Open Science Framework (versus in-text). We have noted in-text that assumption checking information can now be found on the Open Science Framework.

*“All data and a step-by-step guide for our statistical analyses (including data cleaning and assumption checking) are available on the Open Science Framework (*[*https://tinyurl.com/ska2qv9)*](https://tinyurl.com/ska2qv9))*.” p15*

**R1.3** **Pg 16, - Pg 16, Line 350 -352: Please put in () the sentence “ Betas…ska2..”.**

*“However, participant scores for justice sensitivity and disgust sensitivity did not moderate these relationships (betas and confidence intervals for these non-significant interactions are accessible using our step-by-step guide on the Open Science Framework;* [*https://tinyurl.com/ska2qv9)*](https://tinyurl.com/ska2qv9))*.” p15*

**R1.4 Pg 19, Line 381 -383: Please put in () the sentence “ Betas…ska2..”.**

*“Participant scores for justice sensitivity and disgust sensitivity did not moderate any of these interactions (betas and confidence intervals for these non-significant interactions are accessible using our step-by-step guide on the Open Science Framework;* [*https://tinyurl.com/ska2qv9)*](https://tinyurl.com/ska2qv9))*.” p18*

**R1.5 Pg 28, Line 558 -561: Please cut the sentence “ Specifically…things”, as it is sufficient what was already said above.**

We have removed this sentence.

**R2.1 Line 13, on p. 2: The authors should provide some literature evidence to support the assertion that [plastic surgery is increasing in popularity]**

We appreciate the reviewer’s comment. However, authors understand not providing references in the abstract to be best practice. If the editor disagrees, we will include references pertaining to plastic surgery popularity (i.e. as noted in the first paragraph of our manuscript; p3) in our abstract.

**Line 157, on p.8: “First proposed in 2002, the Stereotype Content Model proposes”. Stereotype Content Model should be referenced.**

We have added a reference for this model.

**Line 159, on p.8: The phrase “the way we feel about others is said to depend” should be referenced. Who says this?**

The reference at the end of the next sentence incorporated this statement, but we have now reiterated this reference on the same line.

**Line 174 on p. 9: The word “therefore” is redundant.**

We have removed this word.

**Line 315 on p.15: The word “Results” should be preceded by [The]**

We appreciate the reviewer’s comment but feel the word ‘the’ is redundant in this sentence. However, we are happy to edit the sentence if the editor feels it is necessary.

**Line 326 on p. 16: “They were also were rated…” one [were] is redundant**

We were unable to locate this typo in the text.

**R4.1 My primary concern with the work pertains to the framing of the implications. I think the authors could grapple more with a few specific issues and I believe that doing so would clarify the feminist intentions of the work. As it is presently written, the paper does seem to suggest that women are responsible for the perceptions/stigmatization from others. In particular, that the practical implications of the work suggest intervention for women seeking plastic surgery (e.g., Without this study, uninformed women may choose to seek out plastic surgery and find themselves surprised and disappointed by the negative psychosocial consequences they face postoperatively) rather than intervention for prejudiced audiences. In my reading, this places the burden on the stigmatized group to change, rather than vying for structural change. The authors are not incorrect to suggest that work of the present nature may help to inform women of potential negative outcomes, however, I think the current framing of these implications is slightly inappropriate. To provide a parallel example: it is broadly understood that fat people face significant stigma, however we do not suggest that we should educate people not to get fat so that they will not have to endure that stigma. Broadly, the work would benefit from more in-depth feminist theorization regarding relevant processes of stigma and prejudice.**

We appreciate this comment and agree with the reviewer. We have removed the aforementioned sentence from our introduction and implications are now framed solely as pertaining to better understanding plastic surgery stigma. While we appreciate the reviewer’s suggestion to incorporate further feminist theory and agree it is relevant to the manuscript more broadly, we feel the manuscript would more benefit from an additional discussion of the tangible, real-world implications of stigmatization (as opposed to a discussion of theoretical models). Specifically, we have included a section on the psychosocial outcomes associated with stigma (i.e. “The Negative Impact of Stigma”). We hope this better elucidates some of the implications associated with the present study, and satisfies the reviewer’s concerns.

*“Stigmatized groups face considerable challenges. For example, mental health stigma in the workplace can increase employee’s work-related stress and reduce longevity of employment (19). Similarly, addiction stigma can isolate users from both their social networks and support services (20). Finally, stigmatized sexual minorities are subject to intrusive thoughts and physical symptoms (e.g., diarrhea, faintness, cold, or cough) (21). Thus, there is reason to believe that if plastic surgery is indeed stigmatized, this will adversely impact recipients. Therefore, it is important that we understand whether women who undergo plastic surgery are indeed stigmatized.”p5*

**R4.2 It may be relevant in the introduction to provide plastic surgery statistics specifically for women – men make up an increasing amount of plastic surgery patients, which is important to acknowledge, but also suggests that the statistics presented in the introduction are not particularly relevant to the current study’s focus on women and plastic surgery.**

We have incorporated more women-centric statistics in our introduction.

*“Plastic surgery is particularly popular among women, who account for approximately 87% of all plastic surgery recipients (2). Today, nearly 15 million plastic surgeries per year are performed on women in the US alone – a 169% increase over the past 20 years (3,4). Further, plastic surgery rates increased by more than 20% worldwide between the years 2015 and 2019 (2).” p3*

**R4.3 p.5 “Without this study, uninformed women may choose to seek out plastic surgery and find themselves surprised and disappointed by the negative psychosocial consequences they face postoperatively”. – this is a very strong claim. First, the authors suggest that stigma surrounding plastic surgery is widespread; to make the present claim, the authors should demonstrate that people are not generally aware of this plastic surgery stigma. Second, the authors seem to be suggesting (a) that their findings are generalizable such that they will be relevant to all women seeking plastic surgery, and (b) that these women will encounter their research, allowing it to influence or inform their decisions. This claim should be tempered or preferably removed from the manuscript.**

We agree and have removed this claim from the manuscript.

**R4.4 The authors are very loose with the terminology of non-normative bodies and may wish to reconsider this framing. For example, do people of higher weight have non-normative bodies despite constituting most Americans? Further, the authors’ justification for referring to bodies which have undergone surgery as non-normative is not persuasive; if the body looks similar to others as is suggested, it should not be perceived as non-normative. Relatedly, “higher weight” may not be best terminology here; for example, men typically have “higher weight” than women but are not innately stigmatized as a result. I suggest the authors look to the fat studies literature and indeed adopt the language of “fatness” rather than a euphemism or medicalized terminology. The same applies to the language of “Bigger-bodied” on p.7; this terminology is unclear.**

We define non-normative bodies in our manuscript as “those that don’t align with dominant societal perceptions of how bodies ought to look or be (p8). Given the rampant prevalence of weight stigma in American society, we *do* believe that people who are fat have non-normative bodies for the purpose of this study.

Secondly, we agree that not all plastic surgery bodies can be labelled non-normative. For example, people who do not disclose their plastic surgery to others *and* otherwise don’t ‘look’ like they’ve had surgery might not be perceived as non-normative. However, we feel as though if either one of these conditions is not met (i.e. an individual is known to have had surgery and/or others suspect them to have had surgery because of the way that they look), these bodies *are* subject to be considered non-normative (by our definition of the word). We have amended wording in this section to make this distinction clearer.

*“We therefore posit that those more sensitive to disgust might also express greater plastic surgery stigmatization, given that recipients are also planning to acquire a kind of non-normative body (i.e. one that is is stigmatized because surgically enhanced bodies do not align with perceptions of what bodies ought to be).” p8*

Finally, we agree with the reviewer’s comment on our terminology and have adapted this throughout the manuscript.

**R4.5 Please expand on how perceptions of humanness influence interpersonal relationships (p. 9). If this is important as the authors suggest, the mechanism should be further elucidated.**

*“It is important that we examine humanness in conjunction with the Stereotype Content Model because perceptions of humanness directly influence interpersonal relationships (44). For instance, people who are dehumanized are more often victims of objectification (43) and aggression , and receive less empathy from others (45). Hence, it is imperative that we understand whether plastic surgery recipients are dehumanized.” p9*

**R4.6 Please specify whether gender or sex was measured. Male is used as a descriptor throughout, however, typically when gender is measured the referent would be men. Were participants all cisgender?**

This was an oversight and we have changed terminology throughout our manuscript to reflect gender (e.g., men, women).

**R4.7 Please clarify why the race category for White includes mixed-race people – is there a disadvantage to having a separate category for mixed-race?**

The authors felt that recognising multi-racial participants as members of all racial groups with which they identify would mean participant demographics would be more thoroughly represented in the present study. Further, and perhaps more importantly, we felt it appropriate that participants be given the option to include as many or as few races in their identification as they felt appropriate (i.e. we didn’t want to ‘box’ participants into being either White or mixed-race, if perhaps they identified *predominantly* as White, but also felt alternate categories applied to them). Finally, we presented a free-text options to participants; if they wanted to identify as mixed-race (or anything else that was not listed), they were able to write this in the text box.

**R4.8 The authors should justify why only White women stimuli were chosen, and situate this in a discussion of representation in research. This is particularly relevant given much cosmetic surgery has historically attempted to produce more stereotypically White features.**

We have explained our rationale in the method. Further, we have commented on the limited generalisability of our findings in the discussion.

*“White women stimuli were chosen because we intuited that the majority of our sample would be White.” p11*

*“Speaking further to the generalizability of the study, we note that only White plastic surgery stimuli were used. These findings therefore cannot be generalized to people of color; specifically, we are unable to establish whether plastic surgery recipients who are people of color are subject to the negative plastic surgery effect. Given that plastic surgery has historically attempted to produce more stereotypically White features (e.g., surgeries for the ‘Jewish nose’ or ‘Black nose’), it is especially important that we acknowledge the limited applicability of our findings (28,63).” p24*

**R4.9 Please provide a citation for the Justice Sensitivity inventory, or clarify if this was developed for the present study. If this was developed for the present study, please provide additional information on scale development procedures that were undertaken.**

This was an oversight and we have now included this citation.

**R4.10 Theorizing the link between perceptions of humanness, empathy, and objectification may help to enrich the theoretical setup of the study, particularly given the focus on appearance in the current paper.**

See R4.5

**R.411 p.19 – “the present study demonstrates that negative attitudes toward plastic surgery extend specifically to the act of undergoing plastic surgery itself…” The present study cannot demonstrate this as only intention to undergo plastic surgery was assessed; the act of undergoing it was not. Please rephrase.**

We have rephrased this section and added examples.

*“We posit that exploring plastic surgery as an intended action (versus a completed action) enables researchers to better assess negative perceptions that pertain exclusively to plastic surgery itself, rather than its associated outcomes (i.e. how recipients look after surgery). In other words, studying perceptions of women planning to have plastic surgery allows us to assess shifts in perception regarding recipient character (e.g., “I don’t condone plastic surgery because it is immoral”) as opposed to regarding recipient appearance (e.g., “I don’t condone plastic surgery because I think it makes women look unappealing”). As such, our study aims to establish exactly how a woman’s decision to undergo plastic surgery shapes others’ perceptions of her, irrespective of her surgical outcomes.”p5*

**R6.1 Consequences of perception on women's well-being. Although I understand it might be an issue that deserves to be empirically investigated, I do not think that the authors should include a sentence like "we concluded attractive women seeking cosmetic surgery are subject to experience negative psychosocial outcomes" in the abstract. This is too speculative. Same comment for the part at the end of the Low warmth and Competence paragraph. It might be better to have these hypotheses put at the end of the manuscript in the Implications and Future Directions section.**

We agree with the reviewer. We have reworded the aforementioned sentence in the abstract to better reflect that the authors can only speculate as to how cosmetic surgery influences psychosocial outcomes. We have also reframed the end of our warmth and competence paragraph. Finally, and most significantly, we have noted in our future directions that these outcomes are speculative only, and that future research is needed to confirm whether the negative plastic surgery effect induces contempt and worsened wellbeing.

*“As such, we contend that attractive women seeking plastic surgery may find themselves experiencing negative psychosocial outcomes; they may be interpersonally and professionally affected by others’ feelings of contempt toward them and may also be less likely to fulfil their basic psychological needs. However, we note that at present these outcomes are purely speculative, and that future research is needed to test these associations.” p4*

**R6.2 Attractiveness ratings. Although the photographs were pretested in terms of attractiveness, it could have been better to ask participants to rate the targets’ attractiveness, at least for manipulation check. Moreover, it could have been interesting to see whether planning cosmetic surgery could influence attractiveness ratings and, more importantly, whether it could influence the link between attractiveness and personality inferences.**

We appreciate the reviewer’s feedback. We agree that including a manipulation check would’ve been beneficial and will apply this suggestion to any future research we conduct using these stimuli. However, given that the Chicago Faces Database has previously undergone rigorous and cross-cultural validity assessments, we remain confident that the manipulation was successful (especially considering that we selected the eight most and eight least attractive faces for our stimuli). We also agree that it would be interesting to assess whether cosmetic surgery influences stimuli attractiveness ratings and the relationship between attractiveness ratings and personality inferences. While we are presently unable to conduct this analysis (given that we did not collect attractiveness ratings), we will include this analysis if we conduct similar research in the future.

**6.3 Regarding the gender effect point raised by one reviewer, I understand the authors do not want to include this point in the manuscript because it was not part of their analyses. Still, they could have included the variable as a covariate, as it is done in many research in which gender could have potential effects.**

As previously reported in our first revision letter, with the exception of the single morality outcome, participant gender did not moderate the effect of plastic surgery or participant attractiveness on judgements of the stimuli. Additionally, gender did not have a significant main effect on ratings on our outcome measures. We did not have any theoretical reason to expect gender to play an important role in our study, and given the non-significant impact of gender in our analyses, including it as a covariate would not change any reported results and would, in our opinion, add little to the reported story. We acknowledge that readers may raise a similar question as to the potential impact of gender, and so to address this we have added a sentence to the body of our results summarising the (lack of) gender effects in our study.

*“At the request of a reviewer, we also examined whether any of the aforementioned results were moderated by participant gender. We found that only the relationship between plastic surgery condition and morality but not warmth was moderated by gender, such that the relationship was only significant when participants were women. All other analyses were unaffected by gender and thus it was not included in reported models.” p19*

**6.4 I understood that there were no effects of Justice Sensitivity. But before that, unless I missed it, the authors did not justify the choice of assessing only the Observer's perspective. One could also imagine that the Victim's perspective, for example, could have been a moderator (women feeling less attractive than the target would not want the woman seeking to be more attractive through cosmetic surgery to be even more favored considering that attractiveness can sometimes be a social advantage).**

We agree with the reviewer that other subscales of the Justice Sensitivity Inventory (JSI) could’ve acted as a moderator for the negative plastic surgery effect. However, when conceptualising the study, we ultimately felt that the observer subscale of the JSI subsumed both self-oriented and other-oriented feelings of injustice; for example, items such as “I am upset when someone does not get a reward he/she has earned” can reflect either oneself as ‘someone’ or an external individual as ‘someone’. Conversely, items on the victim subscale (i.e. the subscale noted by the reviewer) solely apply to wrongdoing towards oneself. As such, we chose to use the observer subscale because it incorporated several different possible experiences of perceived injustice and we therefore felt it more comprehensive.

**6.5 The authors mentioned the what-is-beautiful-is-good stereotype considered as the typical example of a halo effect. Do the authors consider that the results t …**

Unfortunately, the second half of this reviewer comment was cut from the email. We therefore weren’t sure how to address this comment.
